# Impact of Animal By-Products on Diet Digestibility and Fecal Quality in Beagle Dogs

**DOI:** 10.3390/life13030850

**Published:** 2023-03-22

**Authors:** Bussarakam Chuppava, Diana-Christin Siebert, Christian Visscher, Josef Kamphues, Amr Abd El-Wahab

**Affiliations:** 1 Institute for Animal Nutrition, University of Veterinary Medicine Hannover, Foundation, 30173 Hannover, Germany; 2 Department of Nutrition and Nutritional Deficiency Diseases, Faculty of Veterinary Medicine, Mansoura University, Mansoura 35516, Egypt

**Keywords:** dog, fatty acids, fecal score, nutrient utilization, particle size, poultry by-product, poultry carcasses

## Abstract

In animal feeds and pet food, meat industry rendered by-products as a source of high-quality protein are commonly used. Among all rendered protein sources, poultry carcasses and neck meal are frequently used as ingredients in commercial pet foods due to their agreeable fatty acid and amino acid profiles, and they have no impact on the palatability of the diet. Nonetheless, it is unclear how poultry by-product meal affects companion animals regarding diet digestibility and fecal quality. This study either aimed to provide information on poultry by-product meal, including coarsely, finely, or very finely ground varieties, regarding their nutrient digestibility and characteristics of feces in dogs. One type of animal by-product meal was used in the three aforementioned particle sizes. Beagle dogs (*n* = 6; body weight, 16.6 kg ± 2.03) participated in a crossover experiment design. Each trial consisted of a five day adaptation period to the diet, and five days of fecal samples were collected and measured for individual apparent nutritional digestibility and fecal scores. The animal by-product supplementation in the diet of dogs was well accepted, with an acceptable percentage of apparent nutrient digestibility. Different particle sizes had no significant effect on the organic matter, crude protein, and crude fat digestibility as well as the fecal fatty acid concentrations. In addition, feces remained firm and well-formed and increased fecal dry matter. This indicates that poultry by-products should be taken into account as a potential dietary protein source in dog food.

## 1. Introduction

Dietary protein is an essential nutrient in canine nutrition [1]. Some parameters, such as digestibility, dietary protein concentration, and composition of amino acids, influence the efficiency of dietary protein utilization in dogs [2], and these characteristics can differ significantly amongst protein sources.

Animal by-products often include optimal levels of amino acids and proteins. However, the nutritive value of these by-products varies greatly between sources, which might be related to various factors, such as particle size, dietary inclusion levels, composition and bioavailability, and processing [2]. In accordance with the Regulations of the European Commission (EC) [3], the animal by-products that may be used as raw materials for pet food also include products from poultry farming and fish processing companies. In the European Union, by-products of the body weight (BW) of cattle, pigs, and chickens account for 46%, 38%, and 32%, respectively, and should be processed suitably [4].

In the EU, around 18 million metric tons were reported yearly, including parts of animals that we do not normally eat (category 3), such as fat trimmings, meat viscera, blood, bones, feathers, hides, and skins, for instance. In addition, out-of-date food products, i.e., former foodstuffs no longer intended for human consumption that may contain ingredients of animal origin (e.g., raw milk, fish or other sea animals, shells of eggs), are used [5,6]. About four million metric tons of animal fats and proteins are produced from these residual materials, with processed animal proteins accounting for approximately 2.5 million metric tons [5]. Animal by-product meals have been (and continue to be) the essential ingredient responsible for the growth and expansion of the global pet food industry since they provide the majority of the protein included in the diets [7]. To move towards a future of sustainability, this considerable amount of material must be handled using methods that are harmless, environmentally responsible, and efficient in terms of the recovery of valuable resources [8]. Thus, animal by-products are produced during the rendering process, which includes heat treatment to render products free of pathogenic microorganisms before subsequent use in animal diets [9]. Moreover, it should also be addressed that using animal by-products in the pet food industry encourages sustainable animal production, as it allows animal protein to be recycled, thus reducing its environmental impact [10].

Although using animal by-product meal as a complete feed for pets is gaining interest, the nutrient digestibility of diets based on this by-product should be considered. Many factors affect diet digestibility [9,11]. Previous studies have reported that the particle size of the raw materials affects nutrient digestibility in monogastric animals [12,13]. The coarse grinding may reduce nutrient exposure to digestive enzymes regarding protein digestibility [13]. Moreover, the degree of grinding could affect the fecal quality in dogs [14]. It is commonly known that grinding is a main concern during food processing [15]. Nevertheless, little information is available about the impact of raw material particle size on carcass by-products fed to domestic dogs as a complete diet. Thus, this study aimed to evaluate and determine the effects of different particle sizes of animal by-product meals in dog food for beagles on the parameters of apparent digestibility of nutrients as well as fecal characteristics.

## 2. Materials and Methods

In accordance with German regulations, the experiments were performed. These animal experiments were approved by the Ethics Committee for Care and Use of Laboratory Animals of the Lower Saxony State Office for Consumer Protection and Food Safety Lower Saxony (LAVES) (approval number: 33.12-42502-04-13/1209).

### 2.1. Study Design and Diet Production

Six healthy intact Beagle dogs (female) with a median age range of six to ten years were obtained from the Institute for Animal Nutrition, University of Veterinary Medicine Hannover, Foundation, Hannover, Germany. Additionally, the study of digestibility is carried out at the Institute for Animal Nutrition, University of Veterinary Medicine Hannover. The dogs’ body weight (BW) was 16.6 kg ± 2.03 at the beginning of the study, and the median body condition score was 4.98 out of 9 over the entire experimental trial, according to Laflamme [16], on a scale from one to nine (values of four or five being considered ideal). A crossover experimental set-up was used. During the feces collection period, the dogs were housed in individual adjacent kennels to enable feces collection. Each trial lasted a total of ten days, beginning with the animal’s adaptation to the meal for five days, then collecting feces for five days to measure individual apparent nutritional digestibility and fecal characteristics.

In the current study, carcasses originating from broilers were obtained from the slaughterhouse, which is located in Lower Saxony, Germany, and used as a complete feed. The broiler carcasses also contained parts of the deep breast muscles and neck, as well as wingtips, poultry fat, and the skull. The carcasses were prepared and milled in three different degrees of grinding (coarse, fine, and very fine) at the Institute for Animal Nutrition, University of Veterinary Medicine Hannover. Particularly in the case of the coarsely comminuted carcasses, parts of the muscles as well as bone components were macroscopically recognizable, while the finely or very finely ground carcasses were distinctly more homogenous and macroscopically resembled a meatloaf. According to NRC guidelines [17], the daily energy requirements of the animals (0.5 MJ ME BW^0.75^/d) based on the prediction equations of maintenance energy requirements for adult dogs by metabolic weight were used to calculate the amount of food to be fed. The dogs in all groups were fed once daily with an amount of 170 g DM/day. The daily amount of food was provided at 08:00 h, the offered amounts and leftovers were weighted, and the intake was recorded. At all times, they had free access to water.

### 2.2. Chemical Analysis

The nutrient composition of the diets as well as fecal samples were determined using protocols developed by the Association of German Agricultural Analytic and Research Institutes e.V. (VDLUFA) [18]. The dry matter (DM), crude ash, crude protein, and ether extract were determined as described by Abd El-Wahab et al. [19]. Briefly, to calculate the DM content, the samples were dried for 12 h (103 °C) and weighed before and after. To measure the crude ash content, the samples were dried and ground before and after combustion for 6 h at 600 °C in the muffle furnace. To determine the total nitrogen content, the DUMAS combustion method and the elemental analyzer Vario Max (CNS Elementar Analysensysteme GmbH, Langenfeld, Germany) were also used, by heating the sample at 1000 °C in a crucible. The crude protein was calculated by multiplying the total nitrogen content by a conversion factor of 6.25. The ether extract was analyzed according to the Soxhlet method via acid digestion (with and without pre-hydrolysis of samples).

The method described in a previous study [20] was used to determine the calcium and phosphorus concentrations. To determine the concentration of calcium, an atomic absorption spectrometer, the Unicam Solaar M Serie atomic absorption spectrometer (Thermo Elemental Ltd., Cambridge, England), was used. While to measure the phosphorus concentration, a photometric characterization based on the vanadate-molybdate method was analyzed by using a UV-Visible Recording Spectrophotometer UV 162 with a wavelength of 356 nm (Schimadzu, Kyoto, Japan).

Ion-exchange chromatography, using the amino acid analyzer LC 3000 (Biotronik, Maintal, Germany), was then used to assess the amino acid content as previously described [19]. The National Research Council (NRC) [17] suggested that the ME content of the diets be calculated based on their chemical composition.

### 2.3. Chemical Composition

Table 1 shows the chemical composition and particle size distribution of the diets. Due to the different particle sizes of the poultry carcass by-product meal ingredient profiles, the results of this study varied markedly. The DM content among the groups was similar (range: 397–407 g/kg fed). The contents of crude ash and crude protein, as well as calcium and phosphorus, were higher in the coarsely ground carcass and neck diet (73.7, 443, 21.7, and 15.2 g/kg DM, respectively), whereas the level of ether extract was lower (485 g/kg DM) when compared to other groups, while the finely ground poultry carcass by-product diet showed a lower crude ash, crude protein, and phosphorus content (52.8, 367, and 9.87 g/kg DM, respectively) but a higher ether extract content (576 g/kg DM). While the very finely ground diet had a higher crude ash, crude protein, and phosphorus content when compared to the finely ground diet.

Regarding the particle size, three different degrees of grinding were analyzed (Table 1). Raw material particle size distribution was independent between diets. The coarse comminution differed most markedly in the particle size >1.00 mm with 45.2%, whereas the fine and very fine ground diets showed a fraction <0.2 mm with 82.3% and 86.1%, respectively.

The amino acid profile of the different particle sizes in the poultry carcass and neck diet is presented in Table 2. Between the different degrees of grinding in the diet, there was a slight variation in the amino acid content. Comparatively high levels of glutamine (coarse: 67.6 g/kg DM, fine: 54.8 g/kg DM, and very fine: 62.1 g/kg DM) were evident, while low levels of cysteine were observed (coarse: 2.90 g/kg DM, fine: 3.10 g/kg DM, and very fine: 3.20 g/kg DM).

### 2.4. Particle Size Analysis

The particle size analysis was determined using the method described by Wolf et al. [21]. The sample, weighing about 30–40 g, was placed into a container with one L of distilled water and mixed for 10 s. After 1 h of soaking, the suspension was then added to a sieve tower consisting of eight analysis sieves (mesh sizes: 3.15, 2.0, 1.4, 1.0, 0.8, 0.56, 0.4, and 0.2 mm, respectively). Ten L of distilled water were used to rinse the sieve tower and put overnight in the drying oven at 103 °C (model 600, Memmert GmbH & Co. KG, Schwabach, Germany) until constant weight was achieved. According to Wolf et al. [21], the individual sieves were then weighed, and a percentage of the total amount of weighed DM was calculated.

### 2.5. Classify Food Acceptance and Apparent Digestibility

The spontaneous acceptance “food intake assessment” (palatability and speed of food intake) was divided into three groups according to Zahn [22]. In summary, a score of 1 indicates the lowest acceptance, a score of 2 indicates a moderate acceptance, and a score of 3 indicates the highest acceptance. The apparent nutritional digestibility was assessed using the entire fecal collection method, according to recommendations of the Association of American Feed Control Officials (AAFCO) [23], which consisted of a five day diet adaptation phase followed by a five day consecutive fecal collection period during which feces were quantitatively collected between 08:00 h and 18:00 h. Fresh feces were collected daily from the concrete floor during the collection period. To measure the DM content, 10% fresh feces per animal/d was determined. Afterwards, the remaining fecal samples (around 90%) were then stored in a freezer at −20 °C for later analysis of the apparent digestibility. At the end of the collection period, the five day fecal samples from each dog were thawed, pooled, and homogenized. It was possible to determine the apparent digestibility (%) for nutrients in the diets by multiplying ((food-feces)/food) by 100 [24].

### 2.6. Fecal Characteristics

During collection, a certain amount of feces was collected every day. The feces were separately collected every 15 min. According to Moxham [25], the five-point scale based on visual appearance is used to assess fecal consistency. The scoring scale runs from 1 to 5, with 1 being extremely firm with small, hard masses; 2 being solid, well-formed “optimum” and consistent stools that do not adhere to the floor; 3 being soft, moist stools, and still-forming feces that retain their shape; 4 being pasty, slushy feces that are unformed; and 5 being watery diarrhea that can be poured. A graphic representation of fecal scores using a 5-point scale has previously been demonstrated by Abd El-Wahab et al. [26]. To determine the dry matter (DM) content, fecal samples were weighed and dried at 103 °C to a constant weight.

To determine the pH level of feces, the samples were mixed with distilled water (1:5 ratio), shaken, left at room temperature for 1 min, and then measured with a pH meter with an accuracy of 0.01, using InLab^®^ Expert Pro (Mettler-Toledo International Inc., Columbus, OH, USA).

### 2.7. Short-Chain Fatty Acid (SCFA)

On the last day of the collection, new feces from each animal were collected to measure the content of fatty acids according to a previous study [27]. To summarize, 1 g of feces was added to safe-lock tubes (2 mL; Eppendorf AG, Hamburg, Germany) and rapidly mixed with an internal standard (10 mL of formic acid at 89 percent and 0.1 mL of 4-methylvaleric acid). The mixture was centrifuged using Megafuge 1.0 (Heraeus Deutschland GmbH & Co. KG, Hanau, Germany) at 4000 rpm for 15 min, and the supernatant was collected. The sample supernatant was analyzed for SCFA (acetic, propionic, iso-butyric, butyric, iso-valeric, and valeric acids). Afterward, gas chromatography was analyzed by using the Unicam Chromatography 610 Series (Kassel, Germany) at 155 °C (with injector at 175 °C and detector at 180 °C); the detector type was flaming ionization; the flow rate was around 0.97 mL/min; and the carrier gas was nitrogen.

### 2.8. Statistical Analysis

The Statistical Analysis System (SAS^®^ version 7.3) was used to determine the mean values and the standard deviation (SD) for all parameters.

Both parametric and non-parametric methods were applied, depending on the distribution analysis of the data. To check the normality first, a Shapiro–Wilk test was used. A Ryan-Einot-Gabriel-Welsch test was used to compare mean values among treatments on the apparent fecal nutrient digestibility, fecal output, DM content, pH, particle size of the feces, and the fecal fatty acid profile. The Kruskal–Wallis test was used for non-normally distributed data, e.g., values in the form of a score. A *p*-value < 0.05 was considered statistically significant.

## 3. Results

The general condition of the dogs was healthy throughout the trial. Body weight and body condition score did not differ between treatments and did not change throughout the study. With regard to the assessment of food intake, high acceptance was observed, thus achieving a high score (range: 3.00), and no significant differences between the diet groups were found. In addition, no food refusals, vomiting, or diarrhea were observed during all the trials.

### 3.1. Apparent Fecal Nutrient Digestibility

The results of the apparent nutrient digestibility of dogs fed the experimental diets are presented in Table 3. No significant differences in the apparent digestibility of nutrients (organic matter digestibility (range: 95.1–95.8%), crude protein (range: 92.8–95.1%), and crude fat (range: 98.3–99.0%)) were observed when dogs were fed coarsely, finely, or very finely ground diets.

### 3.2. Fecal Characteristics

The data on fecal quality are shown in Table 4. In this study, feeding the dogs with grinding degrees did not affect output (range: 14.3–15.7 g DM/d), fecal score (range: 1.50–1.70), or DM content (range: 40.9–43.4%). In addition, the fecal pH level was not different among the groups (range: 6.64–6.83).

Food particle sizes from the different degrees of grinding of the animal by-product meal did not affect the particle size of the feces (Table 4). After feeding the coarsely ground carcass and neck diet, a high proportion of fraction >1 mm was observed (27.1%) compared to the fine and very fine groups (20.8% and 14.5%, respectively). While a high proportion of fraction 0.20–1.00 mm was observed in the feces of dogs fed finely ground by-product meal (24.8%). However, in dogs fed the very finely ground diet, a high proportion of fraction <0.2 mm was observed (65%) compared to the coarse and fine groups (55.0% and 54.4%, respectively).

### 3.3. Short-Chain Fatty Acid in Feces

Table 5 shows the results of the fecal fatty acid concentrations. Dogs fed different particle sizes of animal by-product meal did not affect the concentrations of acetic acid (range: 61.6–64.8), propionic acid (range: 14.0–16.6), iso-butyric acid (range: 2.30–2.50), n-butyric acid (range: 12.8–17.6), iso-valeric acid (range: 3.60–4.20), and n-valeric acid (range: 0.10–0.30).

## 4. Discussion

A special focus was laid on possible structural effects on diet digestibility and fecal characteristics by using differently intensively milled broiler carcasses. In general, during grain processing, grinding is recognized as one of the main concerns [15]. In the current study, the results indicated that the particle size of the poultry by-product meal included in the dog diet did not influence the organic matter digestibility. To the best of our knowledge, fewer studies in dogs have considered the particle size effect of poultry by-products on nutrient digestibility.

It is well-known that high temperatures during the processing systems adversely affect the digestibility of protein in animal by-product meal [28,29]. As reported by FEDIAF (the European Pet Food Industry Federation) [30], the normal digestibility range should be approximately 80%; an investigation of our results in dogs fed either coarse, fine, or very fine diets showed a digestibility of protein within the range of 92.8–95.1%. Interestingly, the particle size seemed to have no effect on the protein digestibility. A previous study by Abd El-Wahab et al. [14] also reported that the particle size or degree of grinding of poultry by-product inclusion in dog food had no effect on the apparent digestibility of crude protein and crude fat. In the present work, when considering particle size, the ether extract was lower in the coarse diet compared to the fine and very fine diets (485 g/kg DM vs. 576 and 547 g/kg DM, respectively). However, we found no difference in apparent crude fat digestibility between treatments (range: 98.3–99.0%). The findings of this study agree with the previous study [31], which described that when feeding the dogs diets containing a high amount of fat (about 320 g/kg DM), the digestibility of fat reached approximately 99%. Nevertheless, our results indicated that the particle size of poultry by-product meal in dog food had no negative effect on apparent nutrient digestibility.

This study provides the results of fecal characteristics in dogs fed diets with different particle sizes of animal by-product meal. The influence of particle size on the fecal characteristics was not apparent in the present study. Fewer studies have considered the effect of varying degrees of grinding for poultry by-products (poultry carcass and neck) on fecal quality in dogs. Fecal scores were closer to the optimal value (score 2) and remained at acceptable levels, with a range of 1.50–1.70 for the poultry by-product meal diet. Our results agree with those reported in a previous study [32], which found that fecal quality was affected by the type and amount of the protein source. The dietary factors influencing the fecal DM content are the digestion and absorption of protein [33]. Nevertheless, in this study, the fecal consistency score was good in the group fed coarsely ground diets with a higher crude protein content (+36–76 g/kg).

The use of the concentration of short-chain fatty acids (SCFA) in feces to assess the activity of the fermentation of the canine colonic microbiota has been practiced for many years [34]. In addition, the canine microbiota have fermentation profiles and activities that are almost identical to those of the transverse colon and the rectum [35]. However, at the large intestine, the SCFA produced is quickly absorbed, up to 95% [36]. Thus, the fecal analysis may not be the best response criterion that reflects the SCFA pattern of the host animal. Our results showed that the fecal SCFA concentrations between different groups did not vary. The distribution pattern of SCFA determined in this study basically agrees with the results of previous studies [37,38]. Van der Steen et al. [37] reported that a high content of protein in the diet related to an increased formation of valeric acid and iso-butyric acid. While valeric acid can be produced at a low concentration and plays a part in the process of fermentation of structural carbohydrates during the decomposition of protein [37]. Nonetheless, including different particle sizes of poultry carcasses and neck meal in the diet had no negative effects on the dogs’ SCFA production. Fermentation results in the production of SCFA, which increases the osmotic pressure of the intraluminal fluid and leads to increased fecal moisture, resulting in reduced fecal dry matter content [39]. With regard to diets based on poultry carcass meal, results are even more variable because of the large variations in the product composition. Meals with low protein but high ash contents lead to considerable losses of minerals, i.e., calcium, phosphorus, and magnesium, via the feces, and, as a consequence, increase the fecal DM content [40].

## 5. Conclusions

For sustainable food, animal production is significantly enhanced by using pet-food-grade animal by-products as feed components. In this present study, it is noted that the use of poultry by-product meal in the dog diets was well accepted. Furthermore, when dogs were fed diets containing coarsely or finely ground poultry carcasses and neck meals, neither the digestibility nor the fecal characteristics were affected. Thus, it was possible to use a poultry by-product meal of either coarse or fine particle sizes in dog diets. However, further studies are still required to investigate the effect of full substitution of poultry carcasses as a complete feed for companion animals in terms of nutritional balance.

## Figures and Tables

**Table 1:** Chemical composition and particle size distribution of the diets with different grinding degrees of animal by-product meal.

Parameter	Unit	Coarse	Fine	Very Fine
Dry matter	g/kg fed	407	402	397
Crude ash	g/kg DM	73.7	52.8	56.6
Crude protein		443	367	407
Ether extract		485	576	547
Calcium		21.7	13.0	12.5
Phosphorus		15.2	9.87	10.0
**Particle size**	%			
>1.00 mm		45.2	8.83	6.56
0.20–1.00 mm		3.30	8.87	7.34
<0.20 mm		51.5	82.3	86.1

DM = dry matter.

**Table 2:** Amino acid levels of the diets with different grinding degrees of animal by-product meal.

Amino Acid	Coarse		Fine		Very Fine	
	g/kg DM	g/100 g CP	g/kg DM	g/100 g CP	g/kg DM	g/100 g CP
Asparagine	33.9	7.72	33.7	9.24	37.9	9.37
Threonine	17.6	4.02	15.2	4.15	18.0	4.46
Serine	17.2	3.93	14.5	3.96	16.1	3.99
Glutamine	67.6	15.4	54.8	15.0	62.1	15.4
Glycine	32.8	7.46	26.6	7.29	27.8	6.88
Alanine	28.2	6.43	23.5	6.44	26.2	6.48
Valine	20.0	4.55	17.8	4.87	20.5	5.09
Cysteine	2.90	0.67	3.10	0.84	3.20	0.80
Methionine	10.4	2.37	9.50	2.59	8.90	2.21
Isoleucine	19.5	4.43	17.0	4.65	19.5	4.84
Leucine	34.4	7.83	28.8	7.89	32.3	8.00
Tyrosine	13.5	3.08	10.9	3.00	12.7	3.14
Phenylalanine	17.5	4.00	15.3	4.18	16.9	4.19
Histidine	11.8	2.69	10.2	2.81	11.6	2.87
Lysine	34.5	7.85	28.8	7.89	32.9	8.15
Arginine	29.6	6.75	22.4	6.13	26.6	6.58
Proline	26.3	5.99	19.0	5.20	20.9	5.18

DM = dry matter. CP = crude protein.

**Table 3:** Apparent nutrient digestibility of selected parameters (in %) of dogs fed diets with various grinding degrees of animal by-product meal (mean ± SD).

Parameter	Coarse	Fine	Very Fine	*p*-Value
Organic matter	95.7 ± 0.42	95.1 ± 0.85	95.8 ± 0.89	0.306
Crude protein	95.1 ± 0.57	92.8 ± 2.32	93.9 ± 1.73	0.207
Crude fat	98.3 ± 0.57	98.4 ± 0.80	99.0 ± 0.22	0.971

No significant differences were noted among treatments, so no superscripts were added.

**Table 4:** Fecal characteristics and fecal particle size of dogs fed the diets with various grinding degrees of animal by-product meal (mean ± SD).

Parameters	Coarse	Fine	Very Fine	*p*-Value
Fecal output (g DM/d)	15.3 ± 1.95	15.7 ± 2.58	14.3 ± 2.08	0.080
Fecal score (1–5)	1.50 ± 0.37	1.70 ± 0.74	1.70 ± 0.72	0.599
DM content (%)	40.9 ± 5.32	41.9 ± 11.1	43.4 ± 11.0	0.878
pH value	6.80 ± 0.45	6.83 ± 0.65	6.64 ± 0.71	0.986
**Particle size (%)**				
>1.00 mm	27.1 ± 11.9	20.8 ± 10.9	14.5 ± 4.61	0.084
0.20–1.00 mm	17.9 ± 4.68	24.8 ± 14.3	20.6 ± 12.3	0.065
<0.20 mm	55.0 ± 13.6	54.4 ± 19.9	65.0 ± 16.4	0.085

No significant differences were noted among treatments, so no superscripts were added. Fecal scores were recorded using a five-point scale (1 = very hard; 2 = solid; well-formed “optimum”; 3 = soft and moist but still-forming; 4 = pasty, unformed feces; and 5 = watery diarrhea).

**Table 5:** Fecal fatty acid concentrations (mmol/kg fresh feces) of dogs fed diets with various degrees of grinding of animal by-product meal (mean ± SD).

Parameter	Coarse	Fine	Very Fine	*p*-Value
Acetic acid	64.8 ± 7.46	61.6 ± 3.04	63.4 ± 10.3	0.535
Propionic acid	16.1 ± 8.41	14.0 ± 4.91	16.6 ± 7.73	0.774
iso-Butyric acid	2.40 ± 0.91	2.50 ± 0.44	2.30 ± 0.95	0.127
n-Butyric acid	12.8 ± 6.05	17.6 ± 5.85	13.4 ± 6.08	0.199
iso-Valeric acid	3.60 ± 1.04	4.10 ± 0.92	4.20 ± 1.77	0.135
n-Valeric acid	0.30 ± 0.24	0.10 ± 0.12	0.10 ± 0.07	0.344

No significant differences were noted among treatments, so no superscripts were added.

## Data Availability

The data presented in this study are available in this manuscript.

## References

[1] Murakami F.Y., de Lima D.C., Menezes Souza C.M., Kaele G.B., de Oliveira S.G., Félix A.P. (2018). Digestibility and palatability of isolated porcine protein in dogs. Ital. J. Anim. Sci..

[2] Case L.P., Carey D.P., Hirakawa D.A., Daristotle L. (2000). Canine and feline nutrition. A Resource for Companion Animal Professionals.

[3] European Commission (EC) (2011). Commission Regulation (EU) No 142/2011 of 25 February 2011 implementing Regulation (EC) No 1069/2009 of the European Parliament and of the Council laying down health rules as regards animal by-products and derived products not intended for human consumption and implementing Council Directive 97/78/EC as regards certain samples and items exempt from veterinary checks at the border under that Directive Text with EEA relevance. Off. J. Eur. Union.

[4] Alm M. Review of the EU feed ban on non-ruminant Processed Animal Proteins. Proceedings of the European Fat Processors and Renderers Association (EFPRA).

[5] Jedrejek D., Lević J., Wallace J., Oleszek W. (2016). Animal by-products for feed: Characteristics, European regulatory framework, and potential impacts on human and animal health and the environment. J. Anim. Feed Sci..

[6] The European Fat Processors and Renderers Association (EFPRA) Glossary for the Animal By-Product Processing Sector. https://efpra.eu/glossary/.

[7] Vanelli K., de Oliveira A.C.F., Sotomaior C.S., Weber S.H., Costa L.B. (2021). Soybean meal and poultry offal meal effects on digestibility of adult dogs diets: Systematic review. PLoS ONE.

[8] Knight R. Livestock & Meat Domestic Data: All Meat Statistics. https://www.ers.usda.gov/data-products/livestock-meat-domestic-data/.

[9] Clapper G.M., Grieshop C.M., Merchen N.R., Russett J.C., Brent J.L., Fahey G.C. (2001). Ileal and total tract nutrient digestibilities and fecal characteristics of dogs as affected by soybean protein inclusion in dry, extruded diets. J. Anim. Sci..

[10] Meeker D.L., Meisinger J.L. (2015). Companion Animals Symposium: Rendered ingredients significantly influence sustainability, quality, and safety of pet food. J. Anim. Sci..

[11] Paßlack N., Galliou F., Manios T., Lasaridi K., Tsiplakou E., Vahjen W., Zentek J. (2021). Impact of the dietary inclusion of dried food residues on the apparent nutrient digestibility and the intestinal microbiota of dogs. Arch. Anim. Nutr..

[12] Wondra K.J., Hancock J.D., Behnke K.C., Hines R.H., Stark C.R. (1995). Effects of particle size and pelleting on growth performance, nutrient digestibility, and stomach morphology in finishing pigs. J. Anim. Sci..

[13] Amerah A.M., Ravindran V., Lentle R.G., Thomas D.G. (2007). Feed particle size: Implications on the digestion and performance of poultry. World’s Poult. Sci. J..

[14] Abd El-Wahab A., Chuppava B., Zeiger A.L., Visscher C., Kamphues J. (2022). Nutrient Digestibility and Fecal Quality in Beagle Dogs Fed Meat and Bone Meal Added to Dry Food. Vet. Sci..

[15] Bazolli R.S., Vasconcellos R.S., de Oliveira L.D., Sá F.C., Pereira G.T., Carciofi A.C. (2015). Effect of the particle size of maize, rice, and sorghum in extruded diets for dogs on starch gelatinization, digestibility, and the fecal concentration of fermentation products1. J. Anim. Sci..

[16] Laflamme D.P., Kuhlman G., Lawler D.F. (1997). Evaluation of weight loss protocols for dogs. J. Am. Anim. Hosp. Assoc..

[17] National Research Council (NRC) (2006). Nutrient Requirements of Dogs and Cats.

[18] Naumann C., Bassler R. (2012). Methoden der Landwirtschaftlichen Forschungs-und Untersuchungsanstalt, Biochemische Untersuchung von Futtermitteln.

[19] Abd El-Wahab A., Chuppava B., Siebert D.C., Visscher C., Kamphues J. (2022). Digestibility of a Lignocellulose Supplemented Diet and Fecal Quality in Beagle Dogs. Animals.

[20] Gerickend S., Kurmies B. (1952). Die kolorimetrische Phosphorsäuerebestimmung mit Ammonium-Vanadat-Molybdat und ihre Nawendung in der Pflanzenanalyse. Pflanzenernähr. Dünger Bodenk..

[21] Wolf P., Rust P., Kamphues J. (2010). How to assess particle size distribution in diets for pigs?. Livest. Sci..

[22] Zahn S. (2010). Untersuchungen zum Futterwert (Zusammensetzung, Akzeptanz, Verdaulichkeit) und zur Verträglichkeit (Kotbeschaffenheit) von Nebenprodukten der Putenschlachtung bei Hunden. Doctoral Thesis.

[23] Association of American Feed Control Officials (AAFCO) (2014). Model Regulations for Pet Food and Specialty Pet Food under the Model Bill.

[24] Kamphues J., Wolf P., Coenen M., Eder K., Iben C., Kienzle E., Liesegang A., Männer K., Zebeli Q., Zentek J. (2014). Supplement zur Tierernährung für Studium und Praxis.

[25] Moxham G. (2001). Waltham feces scoring system-A tool for veterinarians and pet owners: How does your pet rate. Waltham Focus.

[26] El-Wahab A.A., Wilke V., Grone R., Visscher C. (2021). Nutrient digestibility of a vegetarian diet with or without the supplementation of feather meal and either corn meal, fermented rye or rye and its effect on fecal quality in dogs. Animals.

[27] Bunte S., Keller B., Chuppava B., Kamphues J., Visscher C., El-Wahab A.A. (2020). Influence of Fermented Diets on In Vitro Survival Rate of Some Artificially Inoculated Pathogens—A Preliminary Study. Processes.

[28] Kim H., Jung A.H., Park S.H., Yoon Y., Kim B.G. (2021). In vitro protein disappearance of raw chicken as dog foods decreased by thermal processing, but was unaffected by non-thermal processing. Animals.

[29] de Oliveira L.D., de Carvalho Picinato M.A., Kawauchi I.M., Sakomura N.K., Carciofi A.C. (2012). Digestibility for dogs and cats of meat and bone meal processed at two different temperature and pressure levels. J. Anim. Physiol. Anim. Nutr..

[30] The European Pet Food Industry Federation (FEDIAF) (2018). Nutritional Guidelines for Complete and Complementary pet Food for Cats and Dogs.

[31] Hill R.C., Burrows C.F., Ellison G.W., Bauer J.E. (2001). The effect of texturized vegetable protein from soy on nutrient digestibility compared to beef in cannulated dogs. J. Anim. Sci..

[32] Zentek J., Kaufmann D., Pietrzak T. (2002). Digestibility and effects on fecal quality of mixed diets with various hydrocolloid and water contents in three breeds of dogs. J. Nutr..

[33] Weber L.W., Boll M., Stampfl A. (2004). Maintaining cholesterol homeostasis: Sterol regulatory element-binding proteins. World J. Gastroenterol..

[34] Swanson K.S., Grieshop C.M., Flickinger E.A., Bauer L.L., Chow J., Wolf B.W., Garleb K.A., Fahey G.C. (2002). Fructooligosaccharides and Lactobacillus acidophilus modify gut microbial populations, total tract nutrient digestibilities and fecal protein catabolite concentrations in healthy adult dogs. J. Nutr..

[35] Bosch G., Pellikaan W.F., Rutten P.G.P., van der Poel A.F.B., Verstegen M.W.A., Hendriks W.H. (2008). Comparative in vitro fermentation activity in the canine distal gastrointestinal tract and fermentation kinetics of fiber sources. J. Anim. Sci..

[36] McNeil N.I., Cummings J.H., James W.P. (1978). Short chain fatty acid absorption by the human large intestine. Gut.

[37] Van der Steen I., Rohde J., Zentek J., Amtsberg G. (1997). Fütterungseinflüsse auf das Vorkommen und die Enterotoxinbildung von Clostridium perfringens im Darmkanal des Hundes. Kleintierprax.

[38] Minamoto Y., Minamoto T., Isaiah A., Sattasathuchana P., Buono A., Rangachari V.R., McNeely I.H., Lidbury J., Steiner J.M., Suchodolski J.S. (2019). Fecal short-chain fatty acid concentrations and dysbiosis in dogs with chronic enteropathy. J. Vet. Intern. Med..

[39] Bednar G.E., Murray S.M., Patil A.R., Flickinger E.A., Merchen N.R., Fahey G.C. (2000). Selected animal and plant protein sources affect nutrient digestibility and fecal characteristics of ileally cannulated dogs. Arch. Anim. Nutr..

[40] Masuoka H., Shimada K., Kiyosue Yasuda T., Kiyosue M., Oishi Y., Kimura S., Yamada A., Hirayama K. (2017). Transition of the intestinal microbiota of dogs with age. Biosci. Microbiota Food Health.

